# Announcement: 8th International Symposium on Platinum and Other Metal Coordination Compounds in Cancer Chemotherapy ISPCC '99

**DOI:** 10.1155/MBD.1998.174

**Published:** 1998


					8TH INTERNAT YMPOSIUM ON
PLATINUM AND OTHER METAL
COORDINATION COMPOUNDS IN
CANCER CHEMOTHERAPY
ISPCC '99
March 28-31 1999, Oxford, UK
Conference Direc s
L. R. KELLAND I. R. JUDSON
Local Organising Committee
L. ROBERTSON
S. Y. SHARP
F. RAYNAUD
P. WORKMAN
Scientific Committee
M. Abrams
H. Calvert
A. Eastman
N. Farrell
S. B. Howell
S. J. Lippard
F. Muggia
P. Sadler
J. H. Schornnagel
P. A. Andrews
E. Cvitkovic
M. Egorin
T. Hambley
Y. Kidani
G. Los
H. M. Pinedo
J. H. M. Schellens
W. J. F. van der Vijgh
R. Wood
R. Brown
J. C. Chottard
L. C. Erickson
T. Hamilton
J. S. Lazo
M. J. McKeage
J. Reedijk
Z. H. Siddik
E. G. E. de Vries
Preliminary Program
Synthesis & Activity of New Compounds
Drug/DNA Interactions
Molecular Pharmacology/Cell Kill
Platinum Drug Resistance
Preclinical & Clinical Pharmacology
Clinical Trials
Registration
All activities will be hosted by Keble College in the centre of the beautiful historic city of Oxford and
within easy reach of Heathrow and Gatwick airports.
If you are interested in attending this Symposium, please contact
Dr Lloyd R. Kelland
CRC Centre for Cancer Therapeutics, The Institute of Cancer Research
15 Cotswold Road, Sutton SM2 5NG, Surrey, UK
Tel: (44) (0)181 643 8901. Email: Iloyd@icr.ac.uk
The registration fee (approximately 325 for early registration pre-lst December 1998) will cover all
sessions, accommodation and meals.
174

				

